# Domestication affects the structure, development and stability of biobehavioural profiles

**DOI:** 10.1186/1742-9994-12-S1-S19

**Published:** 2015-08-24

**Authors:** Sylvia Kaiser, Michael B Hennessy, Norbert Sachser

**Affiliations:** 1 Department of Behavioural Biology, University of Muenster, Badestrasse 9, 48149 Muenster, Germany; 2 Department of Psychology, Wright State University, Dayton, OH 45435 USA

**Keywords:** adolescence, behavioural development, biobehavioural profile, cortisol, domestication, guinea pig, individual variability, social behaviour, testosterone, wild cavy

## Abstract

Domestication is an evolutionary process during which the biobehavioural profile (comprising e.g. social and emotional behaviour, cognitive abilities, as well as hormonal stress responses) is substantially reshaped. Using a comparative approach, and focusing mainly on the domestic and wild guinea pig, an established model system for the study of domestication, we review (a) how wild and domestic animals of the same species differ in behaviour, emotion, cognition, and hormonal stress responses, (b) during which phases of life differences in biobehavioural profiles emerge and (c) whether or not animal personalities exist in both the wild and domestic form. Concerning (a), typical changes with domestication include increased courtship, sociopositive and maternal behaviours as well as decreased aggression and attentive behaviour. In addition, domestic animals display more anxiety-like and less risk-taking and exploratory behaviour than the wild form and they show distinctly lower endocrine stress responsiveness. There are no indications, however, that domestic animals have diminished cognitive abilities relative to the wild form. The different biobehavioural profiles of the wild and domestic animals can be regarded as adaptations to the different environmental conditions under which they live, i.e., the natural habitat and artificial man-made housing conditions, respectively. Concerning (b), the comparison of infantile, adolescent and adult wild and domestic guinea pigs shows that the typical biobehavioural profile of the domestic form is already present during early phases of life, that is, during early adolescence and weaning. Thus, differences between the domestic and the wild form can be attributed to genetic alterations resulting from artificial selection, and likely to environmental influences during the pre- and perinatal phase. Interestingly, the frequency of play behaviour does not differ between the domestic and wild form early in life, but is significantly higher in domesticated guinea pigs at later ages. Concerning (c), there is some evidence that personalities occur in both wild and domestic animals. However, there may be differences in which behavioural domains – social and sexual behaviour, emotionality, stress-responsiveness – are consistent over time. These differences are probably due to changing selection pressures during domestication.

## Introduction

The development of each advanced civilization was accompanied by the domestication of animals or plants. Hence domestic animals have attended mankind for thousands of years [1, 2]. Most animals living under human control are domesticated. Moreover, domesticated animals play important roles for humans in many aspects of daily life: as pets they are our social companions (e.g. dogs, cats, guinea pigs) and provide protection (e.g. dogs), as farm animals they provide us with food (e.g. meat, milk, eggs) and basic materials (e.g. suet, wax, feather), as laboratory animals they are important for the progress of biomedical research (e.g. mice, rat), and as sporting animals, they even provide us with entertainment (e.g. horses, dogs).

Domestic animals are derived from the wild counterpart by a gradual transformation process over many generations [3–5]. In most cases wild animals have to adapt to human-made conditions, artificial environments and captivity during domestication. This results in long-term genetic changes and finally in the evolution of the domestic phenotype [5–7]. Several forces can influence the evolution of domestic animals: sexual inbreeding and genetic drift of small populations in captivity, relaxed selection with regard to certain pressures, such as predation or resource availability, artificial selection for traits preferred by humans like productivity and fecundity as well as for tameness, and finally “natural selection” in captivity for reduced sensitivity to stress caused by crowding, restriction of movement, parasitism and changes in environment and food sources, which finally leads to adaptation [7–15].

Thus, the conditions under which breeding, care, and feeding of animals are controlled by humans over a period of generations are fundamental for the process of domestication [14–16, 18–20]. This process is always accompanied by distinct changes in morphology, physiology, and behaviour [3, 5, 13, 14, 16–19, 21, 22]. The variability of some characteristics (e.g. body size, colour) is greater in the domestic form [5, 14, 16, 18, 21]. On the other hand, specific domestic characters evolve [13, 23, 24] which are highly convergent between domesticated forms of different species [5, 21], a phenomenon known as domestication syndrome [25]. Together, these domestication characters enable one to readily distinguish between domestic animals and their ancestors.

In this article we will mainly focus on wild and domestic guinea pigs. Domestic guinea pigs are among the few species that are popular pets all over of the world, used as laboratory animals in scientific research and provide a source of meat, particularly in rural populations of South America. In a first step, we will describe how artificial selection shapes biobehavioural profiles during domestication; that is, how wild and domestic animals differ in their appearance, social and sexual behaviours, cognitive abilities, as well as hormonal stress responses. In a second step, we will discuss behavioural development in wild and domestic animals during the early postnatal phase as well as during adolescence; thus, we will highlight during which phases of life differences in the wild and domestic form occur. In a third step, we will address the question of whether or not animal personalities (sensu [26]) exist in the wild and domestic form, and whether or not changes in dimensions of animal personalities occur during the process of domestication.

The review is primarily based on our publications on this topic [27–32]. In addition, some unpublished data [33] are included. In these studies domestic guinea pigs were compared with wild cavies, which were derived from breeding stocks established at the Department of Behavioural Biology, University of Muenster. They originate from lineages belonging to the Universities of Bayreuth and Bielefeld, Germany. In addition, in 1995, wild cavies were captured in the province of Buenos Aires, Argentina, and were introduced in the population of the department. Thus not only were all wild cavies studied born in captivity, but their ancestors had been living in the laboratory for several generations. Our conclusions are discussed in the context of comparisons of other domesticated and wild forms.

## Domestication of the guinea pig

### a) The origin of the guinea pig

The guinea pig (*Cavia aperea* f. *porcellus*) was domesticated approximately 3,000-6,000 years ago in the highlands of South America [5, 34–38]. The Spaniards encountered the guinea pigs in the middle of the 16th century and introduced them into Europe where they rapidly became a popular pet [16, 34, 35, 37]. Nowadays, guinea pigs are one of the most popular pets throughout the world, raised for show and as companions. They also are common laboratory animals in scientific research, used frequently in toxicology, product development and safety testing in the medical field (e.g. [39]).

The main aim of domestication was to provide the indigenous peoples with meat [5]. Even today guinea pigs are one of the main sources of protein in some rural populations of South America. Throughout the years, they have also been used in religious ceremonies and traditional healing practices [5, 16, 34, 35, 37, 38, 40]. In South America guinea pigs are left to scavenge in and around the huts of the natives, and it may be assumed that a similar husbandry has always existed [40, 41].

### b) The wild ancestor *Cavia aperea*

According to anatomical and morphological studies the domestic guinea pig derives from the subspecies *tschudii* of the wild cavy (*Cavia aperea*), which is among the most common and widespread rodents of South America [5, 24, 28, 34–36, 42–44]. The wild cavy is an herbivorous, neotropical species that occurs in humid grassland habitats from Colombia through Brazil into Argentina [41, 45, 46]. Please note, however, that some authors assign the ancestor *tschudii* to its own species, *Cavia tschudii* [47, 48].

In the natural habitat, wild cavies live in single-male groups including up to three females and their offspring [49, 50]. Males do not defend a territory, but they also do not accept other mature males near their females, resulting in little overlap between the home ranges of neighbouring males [49, 50]. Younger and lighter males show alternative strategies as roamers who regularly traverse females’ home ranges or as satellites of males with stable home ranges [50, 51]. The typical habitat of *Cavia aperea* contains a cover zone with high and dense vegetation, which the animals use as protection from predator attacks [43], and an adjacent, more-open zone of short vegetation where cavies forage [50, 52–54].

Under more-restricted semi-natural and laboratory conditions, adult male wild cavies do not tolerate other males, with severe injury or death resulting from agonistic encounters [55]. The male-male competition brings about a polygynous mating system [56]. The higher body mass in males (11% higher than in non-pregnant females) is characteristic of such a mating system. Moreover, the low relative testis weight and the small epididymis size of wild cavies are within the typical range of species with a single-male mating system [56–59]. Female wild cavies are more tolerant of each other, and organize themselves into linear dominance hierarchies. By displaying clear preferences for individual males, females play an active role in bringing about the typical social and mating system for this species [60].

## The biobehavioural profiles of wild and domestic animals in adulthood

### Behavioural aspects

As indicated above, domestic guinea pigs derived from the wild cavy at least 3,000 years ago. From behavioural observations it appears that the repertoire of behavioural patterns is similar in domesticated and wild guinea pigs, as is the case in other domesticated animals and their wild counterparts. Thus, domestication has not resulted in the loss or addition of behavioural elements [27–29, 43, 55].

Distinct differences, however, occur in behavioural frequencies and thresholds ([27–29]; see Table 1): domestic guinea pigs exhibit less aggressive behaviour and more sociopositive behaviour than their wild ancestors. Thus, the process of domestication has led to traits - reduced aggressiveness, increased tolerance of conspecifics - that are typical of other domesticated species (e.g. rats: [61]; cats: Zimmermann cited in [62]; mallard ducks: Desforges and Wood-Gush cited in [11]). This shift in biobehavioural profile of guinea pigs likely developed during domestication because of the immense increase in population densities: wild cavies live in large home ranges from 200 m² up to 1000 m² [49, 50]; domestic guinea pigs, however, can be kept in 6 m² enclosures with up to 20 adult animals [63]. Housing at such high density is probably possible because early breeders of wild cavies chose and selected for the most agreeable individuals, that is those that were least aggressive toward conspecifics as well as humans.

**Table 1:** Endocrinological and behavioural consequences of domestication: Comparison between domestic (Cavia apera f. porcellus) and wild guinea pigs (Cavia aperea).

			Comparison domestic/wild animals	References
**Endocrine parameters**	**endocrine stress response**	Basal cortisol activity	D=W	27,28,29,32
		HPA reactivity (cortisol)	D<W	27,28,29,32
		Basal SAM activity	D=W	28
		(TH activity)		
		SAM reactivity	D<W	27,28,29
		(catecholamines)		
	**gonadal hormones**	Basal HPG activity (testosterone)	D>W	28,32
**Behaviour**	Courtship	Infantile	not exhibited	33
		Adolescent	D>W	32
		Adult	D>W	27,28,29
	Sociopositive	Infantile	D>W	33
		Adolescent	D>W	32
		Adult	D>W	27,28,29
	Aggressive	Infantile	not exhibited	33
		Adolescent	not studied	–
		Adult	D<W	27,28,29
	Attentive	Infantile	D<W	33
		Adolescent	not studied	–
		Adult	D<W	27,28,29
	Vocalization	Infantile	not studied	–
		Adolescent	not studied	–
		Adult	D>W	28
	Exploration	Infantile	not studied	–
		Adolescent	D<W	32
		Adult	D<W	27,28,29
	Risk-taking	Infantile	not studied	–
		Adolescent	D<W	32
		Adult	not studied	–
	Anxiety-like	Infantile	not studied	–
		Adolescent	D>W	32
		Adult	not studied	–
	Play	Infantile	D=W	33
		Adolescent	not studied	–
		Adult	D>W	94
	Learning and memory	Infantile	not studied	–
		Adolescent	not studied	–
		adult	D>W	30,77
	Maternal behaviour towards offspring		D>W	33

HPA = hypothalamo-pituitary-adrenocortical-system; SAM = sympathetic-adrenomedullary-system; HPG = hypothalamo-pituitary-gonadal-system, TH = tyrosine hydroxylase D = domestic guinea pigs, W = wild cavies

Other behavioural changes included an increase in the expression of overt courtship behaviour and in the tendency to vocalize in domestic guinea pigs (Table 1). Furthermore, domestic guinea pigs are less attentive to their physical environment than are wild cavies as indicated by, for instance, the incidence of rearing ([28, 29]; Table 1). This reduction of alertness and sensitivity to environmental change is a further trait typical of domesticated animals [14, 21]. Wild forms of rats [64], dogs [21], pigs [65], and ducks [66] also direct greater attention to the environment than do their domestic counterparts. This is not surprising since a selection against overactive and nervous animals exists during domestication, and sensitivity confers no obvious selective advantage in captivity [67, 68].

Similarly, domestic guinea pigs show less exploratory behaviour than do wild cavies ([29]; Table 1). A decline in exploration seems to be a general character of domestication that is also found in dogs, rats and mice ([69, 70]; but see also [71]). In wild animals, exploratory behaviour is crucial for surviving in their natural habitat [72, 73]: animals have to explore to obtain access to vital resources such as food, water, shelter and mates. However, exploring new environments can be very risky and dangerous. For instance, in the natural habitat of the wild cavy *Cavia aperea* predation can be so severe that mortality rates of up to 50% are observed in a five month period [49]. In a second wild cavy species, *Cavia magna*, very high mortality rates also have been shown [74]. In contrast to this situation in the wild, domestication is characterised by a removal of dangerous and challenging environmental factors [14]. In man-made housing systems, guinea pigs are usually provided with all relevant resources and thus the selection pressure for high levels of exploration and risk-taking is removed.

Concerning learning and memory, Lewejohann et al. [30] tested wild and domestic guinea pigs in the Morris Water Maze, a frequently used test for the assessment of spatial learning in rodents (e.g. in guinea pigs: [75, 76]). Both wild cavies and domestic guinea pigs were able to learn the task. However, male as well as female domestic guinea pigs showed more-rapid acquisition of the task than did their wild conspecifics ([30]; Table 1). In a discrimination task, domestic guinea pigs also performed better than wild cavies [77]. Furthermore the former learned an association and reversal more-rapidly than did the latter [78]. These findings are comparable to those in rats, in which the domestic form shows equivalent or even better performance in learning and memory tasks than their wild ancestor [79]. Thus, artificial selection and breeding does not necessarily lead to degenerated domestic animals with impaired cognitive abilities.

However, one should always be careful in claiming one form as being superior to the other in learning and memory. Performance can depend on the origin of the animals as well as the type of cues used in the tasks. Domesticated and wild gerbils both born in captivity showed similar performance in an auditory discrimination learning task, whereas gerbils caught in the wild performed more poorly [80]. Wild foxes were less able to learn using human gestures as cues compared with domesticated foxes; however, in a control task using non-social cues, the wild foxes were found to be more skilled [81]. A comparison between dogs and wolves revealed that domestication improved performance in animal-human cooperative interactions [82], whereas wolves outperformed dogs in an imitation task: wolves learned quickly to open a box after a conspecific had demonstrated how to succeed; in contrast, dogs were not able to solve the task [83].

### Hormonal aspects

A series of experiments has been conducted to compare the endocrine profile of wild and domestic guinea pigs: while resting, cortisol levels of domestic guinea pigs and cavies in their familiar home enclosure are not different. Wild cavies respond with a larger magnitude increase of their serum cortisol concentrations when exposed to a novel environment than do domestic guinea pigs ([28, 29]; Table 1). Furthermore, serum concentrations of epinephrine and norepinephrine are distinctly higher in the wild than in the domesticated form in response to removal from their homecages ([27–29]; Table 1). Overall, domestic guinea pigs respond to stressors with a smaller response of the hypothalamo-pituitary-adrenocortical (HPA)- and the sympathetic-adrenomedullary (SAM)-systems than their wild counterparts. In addition, significantly lower cortisol levels in response to adrenocorticotropic hormone (ACTH) application indicate a reduction in adrenocortical sensitivity in domestic guinea pigs ([27–29]; Table 1). In general, this lower responsiveness can be regarded as a physiological correlate of the reduced alertness, nervousness, and sensitivity of the domesticated animals compared to their wild counterparts. The lower stress response would seem to be sufficient for domestic animals maintained in artificial housing conditions. Wild animals, however, live in much more challenging environments and thus higher endocrine responsiveness to stressors appears to have evolved for this reason [28]. The activation of each of these systems provides the organism with energy and shifts it into a state of heightened reactivity that is a prerequisite for responding to environmental challenges in an appropriate way. Finally, guinea pigs have higher basal plasma testosterone levels than do wild cavies ([28]; Table 1). As mentioned above, guinea pigs also show higher levels of courtship behaviour. There might be a causal relation between higher frequencies of courtship behaviour and higher testosterone concentrations in guinea pigs though the direction of this putative relation is unclear. That is, social interactions including courtship behaviour can result in increased testosterone titers [84–87] and elevated testosterone can increase courtship behaviour [88, 89].

## Development of the biobehavioural profile in the wild and domestic form

In most studies investigating domestication effects, adult animals of the wild and domestic form are compared. Thus, the question arises as to whether differences found in adult animals are already present in earlier phases of life. Here we summarize findings from comparisons of domestic and wild guinea pigs during the early postnatal phase as well as during adolescence, i.e. before and shortly after sexual maturity.

### Early postnatal phase

Wild and domestic guinea pigs are highly precocial. They are able to feed on solid food and locomote from shortly after birth [90, 91]. Accordingly, maternal care is limited mainly to lactation and grooming. Remarkably, domestic females suckle their male and female offspring significantly longer than do wild females in comparable environments with the same diet available, suggesting increased maternal care in the domestic form ([33]; Table 1).

Sabaß [33] recorded the behaviour of male and female wild and domestic guinea pigs on day 11, 15 and 19 after birth, that is, up to shortly before weaning which occurs at about 21 days of age. Aggressive as well as courtship and sexual behaviour were only rarely shown by infant animals, and no differences in these behaviours could be found between the two different forms (Table 1). Significant differences occurred, however, for sociopositive behaviour and attentiveness (Table 1): Infant male and female domestic guinea pigs showed longer duration of *bodily contact* with their parents than did infant wild cavies at all three observation days, and infants of the domestic form were less attentive to their environment than same-aged wild cavies. Thus, these findings during the early postnatal phase of life replicate the differences described in adult animals (see above; [28, 29]). Interestingly, comparable amounts of play behaviour were shown by infant wild and domestic animals ([33]; Table 1). In this species play is primarily solitary and consists of *frisky hops* (executing upward leaps and turning the head or foreparts sharply while in the air) and *run off* (starting with a short and fast run, then stopping suddenly and changing direction). Generally, it is assumed that play is important for developmental processes [92] by, for example, stimulating muscle growth [93]. If there are similar requirements for these developmental processes for pups of both the domestic and wild form, there might be a similar selection pressure on young wild and domestic animals to play during early phases of life. In adulthood, however, male guinea pigs play more often than male wild cavies ([94]; Table 1). Other domesticated animals, such as dogs and cats exhibit apparent play in adulthood, whereas their wild ancestors play only at younger ages [95], Sambraus 1978 and Stauffacher 1990 cited in [96]. During the process of domestication, animals typically live under conditions in which predators are rare or absent and important resources are sufficiently available; that is, domesticated animals mainly exist in relaxed, non-stressed situations over generations. Generally, play occurs only in such situations. Thus, the threshold for play behaviour in domesticated adults might be reduced. Another explanation is that domestication results in retarded behavioural development [94, 95, 97], or in the retention of juvenile features into sexual maturity, a phenomenon known as neoteny [98]. It may be that the more frequent display of play behaviour in adult domestic guinea pigs in comparison to the wild form is a sign of neoteny.

### Adolescence

In a recent study, we have compared the biobehavioural profile in domestic guinea pigs and wild cavies from early to late adolescence [32]. Three different domains of the biobehavioural profile were investigated: anxiety-like and risk-taking behaviour, social and courtship behaviour as well as cortisol stress responses. To assess anxiety-like behaviour, the animal was placed into an open arena (open-field test), and the percentage of time that the individual spent away from the walls in the central area was recorded. As a further measure of anxiety-like behaviour, the latency to leave a dark box, and the percentage of time spent in a light area, were recorded in the dark-light-test. Risk-taking behaviour was measured in the step-down-test, in which the animal is placed on an elevated platform and the latency to step down is recorded. Social and courtship behaviour were assessed in two tests in which the animals were either introduced to an unfamiliar infant or an unfamiliar non-oestrous female. In these tests the latencies to approach the unfamiliar individuals and the frequencies of contact and courtship behaviour, respectively, were recorded. Finally, stress reactivity was assessed by placing the animals singly into an unfamiliar enclosure and by determining serum cortisol concentrations at the beginning of each test (basal values) as well as 1, 2 and 4 hours later. All tests were conducted twice: during early adolescence (at about 50 to 60 days of age) that is, before reaching sexual maturity - which occurs around 75 days of age - as well as during late adolescence (at about 120 to 130 days of age).

**Figure 1:**
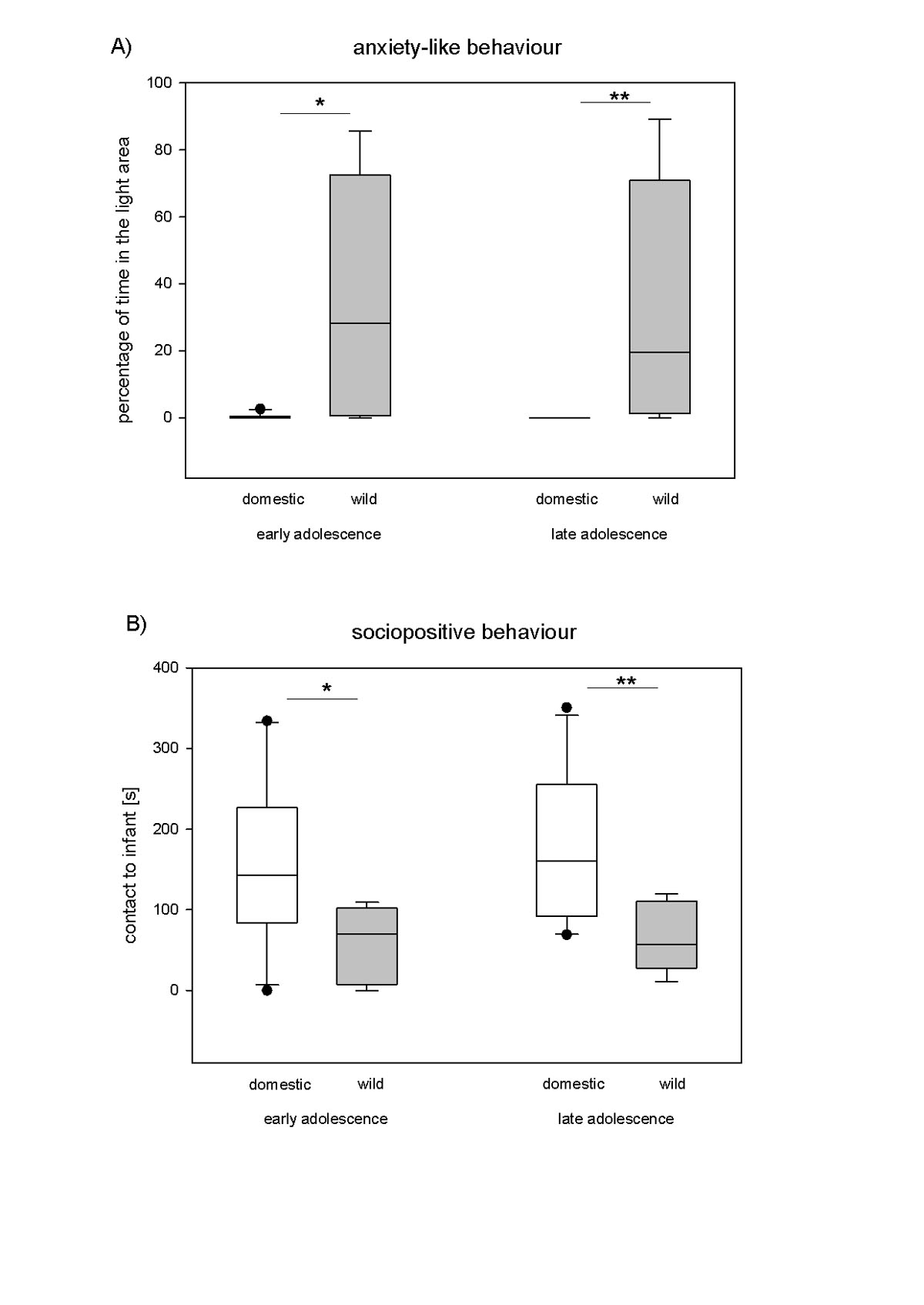
Behaviour of domestic guinea pigs and wild cavies in one test of anxiety-like behaviour (A) and one test of social behaviour (B) during early and late adolescence. A): percentage of time spent in the light area of the dark-light test. B): time spent in contact with an unfamiliar infant in a male/infant interaction test. Data are shown as medians, 10th, 25th, 75th, and 90th percentiles; outliers are indicated by dots. Statistics: Mann–Whitney U-test (two-tailed), N_Domestic_ = 10, N_Wild_ = 8; * = p ≤ 0.05; ** = p ≤ 0.01. Redrawn after [32].

Early and late adolescent domestic guinea pigs showed more anxiety-like behaviour in the open-field and dark-light test in comparison to the wild form (Figure 1A; Table 1). Furthermore, domesticated animals were less likely to take risks in the form of descending from the elevated platform (Table 1). Regarding social behaviour, early and late adolescent male guinea pigs directed more social activity towards unfamiliar females and infants than did same-aged male wild cavies (Figure 1B; Table 1). Finally, the cortisol response to a novel environment was significantly higher in early and late adolescent wild cavies compared to early and late adolescent domestic guinea pigs. In contrast, basal values of cortisol reflecting conditions in the familiar home enclosure did not differ between the wild and domestic form (Table 1). Basal testosterone concentrations were markedly higher in guinea pigs than wild cavies in early as well as late adolescence (Table 1). As referred to earlier, the latter result may be related to the increased levels of courtship and sexual behaviour, which frequently are found in domesticated animals [14, 28]. In summary, the comparison of wild cavies and domestic guinea pigs from early to late adolescence replicate the results obtained in earlier studies of adult animals (see above).

## Effects of domestication on animal personality

Biobehavioural profiles may vary conspicuously between members of the same species. Understanding such variation is of major importance because it is frequently related to differences in reproductive success, susceptibility to disease and quality of life [99]. If an individual biobehavioural profile is consistent over time and/or across contexts, it is often described as “animal personality” [26, 100, 101]. An ever increasing number of reports show that animal personalities are widespread across a great variety of taxa, including fish, birds and mammals [100, 102, 103], and even invertebrates [104].

It would be of interest to know whether or not the same behavioural and physiological traits are stable over time and/or across context in the domestic form as compared to the wild ancestor. To our knowledge, there are no studies in this area that directly compare the domestic and wild forms. However, Zipser et al. [31] recently published a study regarding animal personalities in domestic guinea pigs and Guenther and Trillmich [105] provided data for the wild cavy.

**Figure 2:**
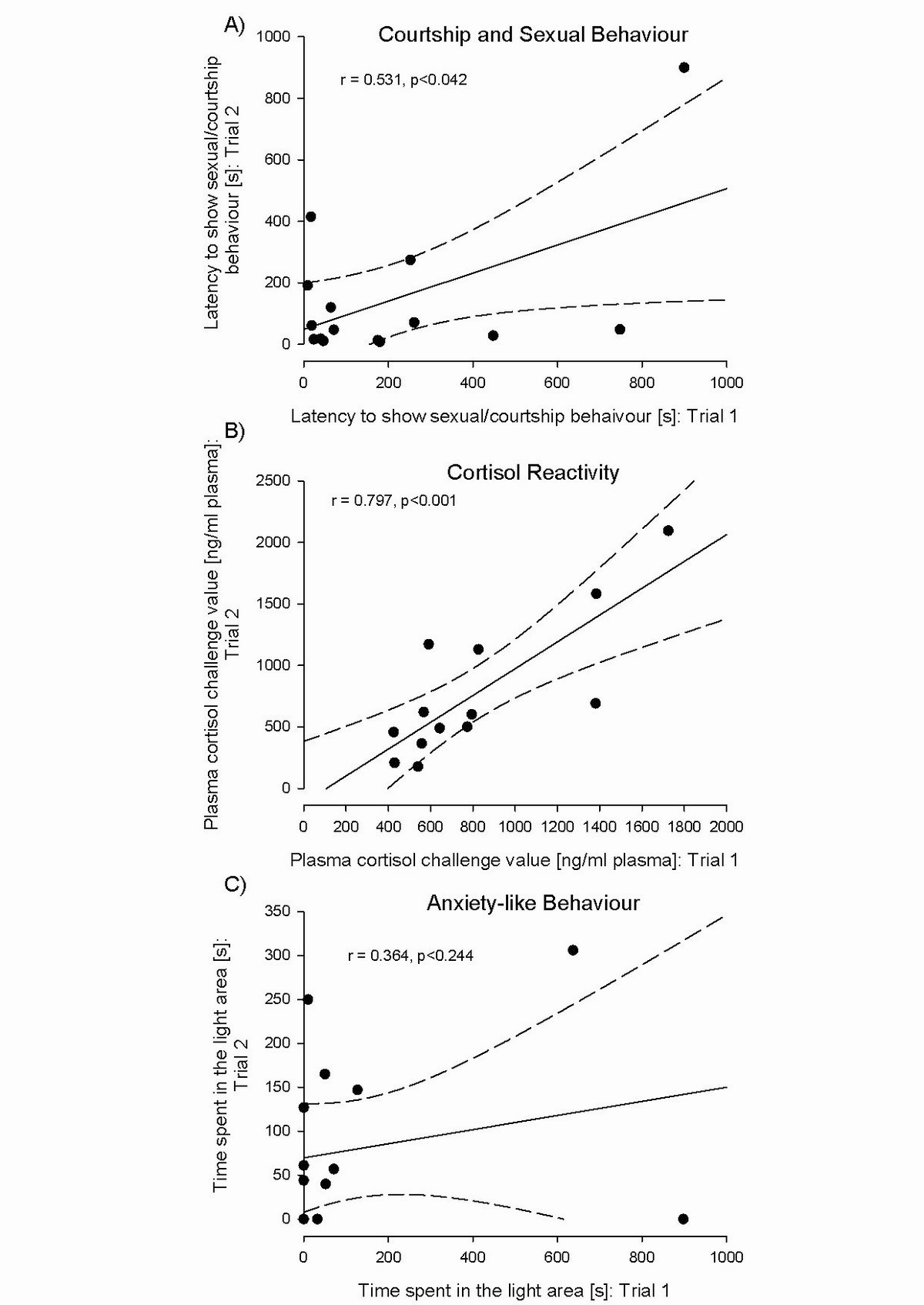
Correlation of biobehavioural traits over time (8 weeks). A) Latency of sexual and courtship behaviour (*intensive anogenital licking, rumba, mounting, pelvic thrust*) when exposed to an unfamiliar non-receptive female. B) Plasma cortisol response after exposure to a novel enclosure (cortisol-reactivity test: 2h reaction value). C) Time spent in the light area of the dark-light test. Dots represent single individuals. Statistics: Pearson's product-moment correlation, A, C: N=15, B: N=13. The 95% confidence interval (dashed lines) is also shown. Redrawn after [31].

In domestic guinea pigs, Zipser et al. [31] investigated the temporal stability of personality traits in adult males, namely courtship and sexual behaviour displayed with an unfamiliar, non-oestrous female, risk-taking as well as anxiety-like behaviour in novel environments and cortisol-stress reactivity in a challenging situation. The males were 7 to 18 months old and were tested twice at an interval of 2 months. Sexual and courtship behaviour displayed a clear consistency over time. The more sexual and courtship behaviour a male exhibited during the first test, the more he showed during the second (Figure 2A). This agrees with findings in pioneering work on guinea pigs by Young and colleagues [106, 107], in which males were exposed to an unfamiliar oestrus female and a sex drive score was calculated from the courtship and sexual behaviour displayed. When tested repeatedly over time, highly stable sex drive scores were found. After castration, sexual behaviour declined strongly in all males, but was restored by experimental androgen replacement. Remarkably, after androgen replacement therapy the males’ sex drive scores returned to their individual pre-castration levels, irrespective of the dosage of androgen [106].

Stress reactivity in domestic guinea pigs exposed to a novel enclosure showed substantial individual variation. As was the case for social behaviour, this individual stress reactivity was very stable over time. The higher the cortisol response during the first challenge test, the higher the response when tested for the second time about 2 months later (Figure 2B; [31, 108]). It appears that such clear individual stability of cortisol responsiveness over time has only rarely been shown in an animal model (for a further example in tree shrews see [109]).

In contrast to social behaviour and acute stress responsiveness, no consistency was found for emotional behaviour: Neither anxiety-like nor risk-taking behaviour proved to be stable over time (Figure 2C; [31]). It is somewhat surprising then that in the Guenther and Trillmich [105] study investigating anxiety-like and risk-taking behaviour in wild cavies the opposite conclusion was drawn. In that study an open-field-test, a long-field and a novel-object test were conducted. In the long-field test, a 5-m long corridor was attached to the standard housing enclosure so that the animals could freely explore the new environment. In the novel object test, an unfamiliar object such as a red plastic toy pig was introduced into the homecage. Parameters such as distance traversed in the open-field-test, latency to initiate exploration in the long-field test and to contact the novel object were used to estimate anxiety-like reactions and risk-taking. In contrast to domestic guinea pigs, individual emotional behaviour in terms of latency to explore in the long-field-test as well as of distance explored in the open-field-test was stable over time in the wild ancestor.

How might this difference between the wild and domestic form be reconciled? Current theories on the emergence of personality traits emphasize the importance of unpredictable environments [101, 110, 111]. In especially uncertain or ever-changing environments, one strategy would be for individuals to continually change their behavioural responses to adjust to the environmental changes. This strategy is not only very costly it also involves the risk that appropriate behavioural adjustment will repeatedly lag behind environmental change. In such situations, it may be a better strategy to develop stable traits of behavioural responses which may be inappropriate in some situations, but are effective in most [101]. This line of reasoning may help to account for the differences in personalities of wild cavies and domestic guinea pigs. In the natural habitat of wild cavies, the environment is rather unpredictable due to heavy predation pressure and tremendous fluctuations of population densities [49, 50]. In this situation stable emotional traits seem to be adaptive. Because these environmental influences are removed during domestication [14], stable emotional responses are no longer necessary, and thus may be lost in domestic guinea pigs [32].

## Conclusion

Domestication is a complex evolutionary process bringing about significant changes in biobehavioural profiles. There is growing evidence that the differences in behavioural and endocrine traits between domestic and wild animals of the same species reflect the different demands of the natural habitat in which wild animals are living and of the man-made artificial conditions to which domestic animals are exposed [28, 29, 32]. Compared to domestic animals, the wild ancestor is generally characterized by greater exploration and risk-taking as well as less anxiety-like behaviour. These behavioural patterns presumably help the animals to cope with the ever changing and fluctuating conditions of the natural habitat. Furthermore, animals of the wild form are characterized by more-vigorous stress responsiveness. Since the increase of glucocorticoids and catecholamines ultimately provides the animal with more energy, it seems likely that robust responsiveness of the stress hormone systems is a prerequisite for coping successfully with the demands of the ecological niche, e.g., high predation pressure. In contrast, domestic guinea pigs are more sociable and less aggressive. These traits facilitate survival in the dense housing conditions in which domestic animals often are maintained. Domestic guinea pigs also explore less and take fewer risks, probably because in man-made housing conditions all relevant resources such as food are available. Finally, the domestic form shows diminished stress responsiveness. This trait may be regarded as an adaptation since the less-challenging housing conditions do not require excessive energy expenditure and thus robust stress responsiveness may be wasteful rather than valuable. A further domestication character is reduction in brain weight [1, 80, 112]. In guinea pigs for example brain weight is reduced by about 13% in comparison to wild cavies [112]. For many years, it was assumed that this trait was accompanied by reduced cognitive abilities. Recent studies comparing wild and domestic animals, however, suggest that domestic animals are not inferior with respect to memory and learning.

In most studies investigating domestication effects, adult animals of the domestic and wild form are compared. Here we show that differences between the biobehavioural profiles of guinea pigs and their wild ancestor are already present during early stages of life, i.e. during the early postnatal phase as well as early and late adolescence. While differences between the domestic and wild form almost certainly involve genetic alterations brought about by artificial selection during the process of domestication [113], they may also be due to environmental conditions and social experiences during the prenatal and perinatal phase that differentially influence brain development in domestic and wild forms [114]. Such early influences leading to different biobehavioural profiles were shown to be mediated by epigenetic effects, which can be stable over generations [115–118].

Finally, we provide evidence that personalities occur in both wild and domestic animals. However, there appear to be differences in which behavioural domains are stable over time. In domestic guinea pigs, social and sexual behaviour as well as cortisol stress-reactivity show good temporal stability, whereas emotional behaviour does not. This contrasts with the wild ancestor in which emotional behaviour does appear stable over time. These initial findings on the effect of domestication on animal personality are intriguing, though much remains to be learned. This is, perhaps, one of the most fertile areas for future research on the domestication process.

## Declarations

We acknowledge financial support for this publication by the German Science Foundation (FOR 1232) and the Open Access Publication Fund of Bielefeld and Muenster University.
